# Induction of stem-like cells with malignant properties by chronic exposure of human lung epithelial cells to single-walled carbon nanotubes

**DOI:** 10.1186/1743-8977-11-22

**Published:** 2014-05-11

**Authors:** Sudjit Luanpitpong, Liying Wang, Vincent Castranova, Yon Rojanasakul

**Affiliations:** 1 Pharmaceutical and Pharmacological Sciences Program, West Virginia University, Morgantown, WV 26506, USA; 2 Mary Babb Randolph Cancer Center, West Virginia University, Morgantown, WV 26506, USA; 3 Pathology and Physiology Research Branch, National Institute for Occupational Safety and Health, Morgantown, WV 26505, USA

**Keywords:** Carbon nanotubes, Stem cells, Lung epithelial cells, Tumorigenesis, Malignant transformation

## Abstract

**Background:**

Carbon nanotubes (CNT) hold great promise to create new and better products for commercial and biomedical applications, but their long-term adverse health effects are a major concern. The objective of this study was to address human lung cancer risks associated with chronic pulmonary exposure to single-walled (SW) CNT through the fundamental understanding of cellular and molecular processes leading to carcinogenesis. We hypothesized that the acquisition of cancer stem cells (CSC), a subpopulation that drive tumor initiation and progression, may contribute to CNT carcinogenesis.

**Methods:**

Non-tumorigenic human lung epithelial cells were chronically exposed to well-dispersed SWCNT for a period of 6 months at the physiologically relevant concentration of 0.02 μg/cm^2^ surface area dose. Chronic SWCNT-exposed cells were evaluated for the presence of CSC-like cells under CSC-selective conditions of tumor spheres and side population (SP). CSC-like cells were isolated using fluorescence-activated cell sorting and were assessed for aggressive behaviors, including acquired apoptosis resistance and increased cell migration and invasion *in vitro*, and tumor-initiating capability *in vivo*. Non-small cell lung cancer cells served as a positive control.

**Results:**

We demonstrated for the first time the existence of CSC-like cells in all clones of chronic SWCNT-exposed lung epithelial cells. These CSC-like cells, in contrary to their non-CSC counterpart, possessed all biological features of lung CSC that are central to irreversible malignant transformation, self-renewal, aggressive cancer behaviors, and *in vivo* tumorigenesis. These cells also displayed aberrant stem cell markers, notably Nanog, SOX-2, SOX-17 and E-cadherin. Restored expression of tumor suppressor p53 abrogated CSC properties of CSC-like cells. Furthermore, we identified specific stem cell surface markers CD24^low^ and CD133^high^ that are associated with SWCNT-induced CSC formation and tumorigenesis.

**Conclusions:**

Our findings provide new and compelling evidence for the acquisition of CSC-like cells induced by chronic SWCNT exposure, which are likely to be a major driving force for SWCNT tumorigenesis. Thus, our study supports prudent adoption of prevention strategies and implementation of exposure control for SWCNT. We also suggest that the detection of CSC and associated surface markers may provide an effective screening tool for prediction of the carcinogenic potential of SWCNT and related nanoparticles.

## Background

Carbon nanotubes (CNT) are a major class of engineered nanomaterials that are being produced on a massive scale for a wide range of industrial and biomedical applications. Their rapid growth in utility is attributed to their unique properties such as light weight, high tensile strength, conductivity and flexibility [1,2]. The global market for CNT is estimated to reach trillion dollars in the next decade [3], and so will the increase in human exposure during manufacturing, consuming, and disposal. Despite this growing trend, their adverse health effects, especially long-term health effects, are relatively unknown. CNT share several properties (e.g. high aspect ratio, durability, and biopersistence) and route of exposure (e.g. respiratory) with asbestos fibers [4-6], which are known human lung carcinogens. Thus, CNT exposure may cause heath consequences similar to asbestos exposure, which include lung cancer and mesothelioma.

The lungs are the major target organ for airborne CNT exposure. CNT have been shown to migrate into the alveolar interstitial compartment where the clearance rate is low [7-9]. Recent animal studies have shown that inhaled CNT that penetrate lung tissue could persist in the lungs 6 months post-exposure [10]. Such biopersistence and chronic interaction with lung epithelial cells could potentially lead to carcinogenesis [10,11] since biopersistence is a critical factor in the paradigm of hazardous fibers and is a basis for the classification of their carcinogenic potential [12,13]. The acute effects of high-dose CNT have been widely studied. *In vitro*, CNT can induce apoptosis, DNA breakage, multipolar mitosis, and activation of key molecular events involved in carcinogenesis, e.g. MAPK, AP-1, NF-κB, and Akt [14-18]. In animals, a single intraperitoneal injection of multi-walled (MW) CNT in heterozygous p53 mice caused asbestos-like mesothelioma [19,20], while their short-term intraperitoneal instillation in C57BL/6 mice induced granuloma formation [21]. Short-term inhalation of single-walled (SW) CNT was shown to trigger mutations of K-*ras* gene locus in the lung of C57BL/6 mice, which is a common event observed in lung tumors [22].

Unlike their acute effects, the chronic effects of CNT have not been well addressed due to technical difficulties and limited experimental models. Carcinogenesis is a multi-step process requiring long-term exposure to the carcinogens. Typical developmental period for fiber-induced lung cancer in humans is 30–40 years [23]. To mimic this long-term carcinogenic process, we have recently developed a chronic exposure model in which human lung bronchial and small airway epithelial cells, a major cellular target of human lung carcinogenesis, were continuously exposed to low-dose, physiologically relevant concentrations of SWCNT for a prolonged period of 6 months. Such chronic exposure resulted in irreversible malignant transformation and aggressive behaviors of the cells, activation of cancer-related canonical pathways, and induction of tumorigenesis in a mouse model [24,25]. A similar induction of aggressive/invasive phenotype was observed in mesothelial cells chronically exposed to SWCNT [26]. However, the fundamental mechanisms of SWCNT tumorigenesis are unclear at present.

Evolving research indicates that cancer stem cells (CSC) are a potential driving force of tumor initiation and progression due to their self-renewal and unlimited proliferative capacity [27,28]. The existence of CSC was reported in human cancers, including brain, breast, bone marrow, prostrate, colon, and lung [29,30]. The present study was undertaken to investigate whether chronic SWCNT exposure can induce lung CSC, and whether these cells possess tumorigenic activity. Our data demonstrated for the first time that SWCNT can interact with lung epithelial cells to induce CSC which have the propensity to form tumor spheres, indicating their neoplasticity and self-renewal capacity. Concurrent studies have shown that a small subpopulation of cells characterized as side population (SP) may be a source of CSC [30,31]. Here, we report the presence of this distinct SP subpopulation in chronic SWCNT-exposed lung cells that is enriched with CSC and shows more aggressive cancer phenotypes and tumor-initiating capability as compared to non-SP (non-CSC). These CSC also exhibit several stem cell phenotypes, including self-renewal and regeneration, and express a high level of pluripotent stem cell markers. Together, our study strengthens the earlier finding on potential SWCNT carcinogenicity and unveils a novel mechanism of SWCNT tumorigenesis toward the path of acquiring CSC traits, which may be shared by other engineered nanotubes and nanofibers.

## Results

### CNT characterization and dosage calculation

SWCNT were obtained from Carbon Nanotechnology (CNI, Houston, Texas) and were purified by acid treatment to remove metal contaminates. Elemental carbon analysis by NIOSH Manual of Analytical Methods (NMAM 5040) and metal analysis by nitric acid dissolution and inductive coupled plasma-atomic emission spectrometry (ICP-AES) showed that the purified SWCNT contained 99% elemental carbon and less than 1% of contaminants. The metal residues were mostly iron (Fe) at 0.23% by weight. The Brunauer Emmet Teller (BET) surface area, length (L), and width (W) of individual dry SWCNT were 400–1040 m^2^/g, 0.1-1 μm (L), and 0.8-1.2 nm (W) (*see* Table 1 for summary). SWCNT were dispersed by acetone/sonication method as previously described [16,24,32]. The CNT doses used in the *in vitro* exposure studies were calculated based on *in vivo* CNT exposure data normalized to alveolar surface area in mice. For example, the lowest dose which induced positive *in vivo* biological response was 10 μg/mouse lung (0.5 mg/kg body weight) [8,9]. Dividing this dose by the average alveolar surface area in mice (~500 cm^2^) [33] indicates the *in vitro* surface area dose of 0.02 μg/cm^2^, which is equivalent to a human lung burden for 8 hours/day over a month at 400 μg/m^3^ (high CNT level reported in a research facility) [34] or about 3 years at 10 μg/m^3^ (average CNT level in U.S. facilities) [35].

**Table 1:** Physicochemical properties of particles used in this study

	**SWCNT**	**Crocidolite asbestos**
Manufacturer	CNI	NIEH, Kalahari Desert
Catalog reference	Unidym™	CAS 12001-28-4
Synthesis	HiPco	Natural
Purification	Acid treatment	-
BET surface area (m^2^/g)	400-1040	9.8
Dry mean length (L) (μm)	0.1-1	210
Dry mean width (W) (nm)	0.8-1.2	10
% carbon (w/w)	> 99%	-
% metal impurity (w/w)		
Fe	0.23%	31.8%
SiO_2_	-	50.9%
Other trace metals	Not detectable	6.2%

### Chronic SWCNT exposure induces CSC-like cells

**Figure 1:**
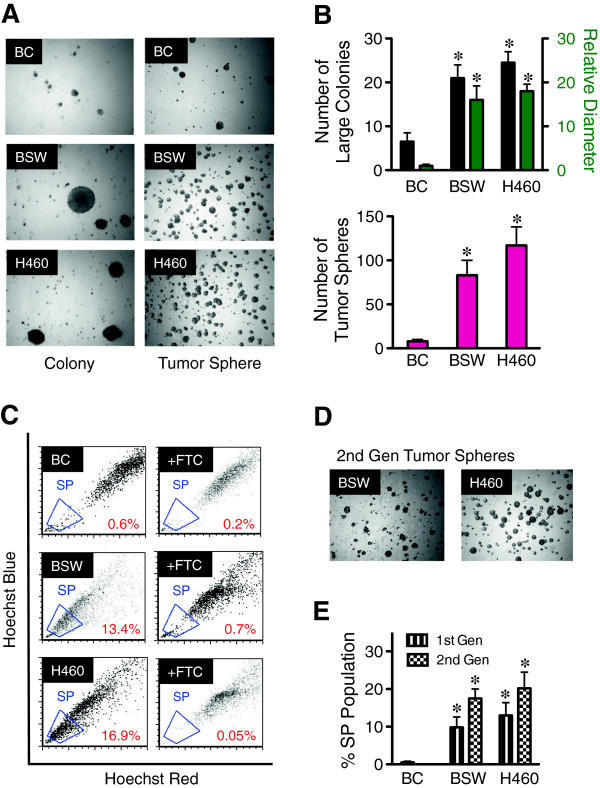
**Chronic exposure to SWCNT induces malignant transformation and CSC formation in bronchial epithelial cells. (A)** Analysis of colony formation (*left*) and tumor spheres (*right*) in passage control BC cells, chronic SWCNT-exposed BSW cells, and lung cancer H460 cells after two weeks of culture. **(B)** Quantitative analysis of colony (*top*) and tumor spheres (*bottom*). **(C)** Analysis of side population (SP) in BC, BSW, and H460 cells in the presence or absence of fumitremorgin c (FTC) using FACS. SP cells (*box*) are determined by their disappearance in the presence of FTC. **(D, E)** Analysis of second-generation tumor spheres (*D*) and SP (*E*). Data are mean ± SD (*n* = 4). *p < 0.05 *vs*. control BC cells.

Subconfluent cultures of human small airway epithelial cells (SAEC) and bronchial BEAS-2B epithelial cells were continuously exposed to a low-dose physiologically relevant concentration of SWCNT at 0.02 μg/cm^2^ in culture and passaged weekly for a period of 6 months. The cells were cultured in normal medium without SWCNT for at least ten passages prior to further experiments. These chronic SWCNT-exposed cells were previously shown by our group to possess irreversible malignant transforming properties, altered cancer-related canonical pathways, and tumor forming activity in mice [24,25]. The ability to self-renew and generate different differentiated progeny is fundamental properties of CSC. A key characteristic of CSC is the formation of tumor spheres in stem-cell selective condition of serum-free media and non-adherence [31,36]. To first assess the existence of CSC in SWCNT-transformed cells, chronic SWCNT-exposed bronchial epithelial cells (designated as BSW) and passage-matched control (BC) cells were evaluated by the tumor sphere formation assay as well as soft-agar colony formation assay which is the most stringent indicator of malignant transformation [37]. Chronic SWCNT-exposed BSW cells formed large (>50 μm) colonies and tumor spheres, similarly to the positive control non-small cell lung cancer H460 cells, whereas the passage control BC cells showed minimal number of small colonies and spheres (Figure 1A and B), indicating malignant transformation, neoplasticity, and self-renewal property of SWCNT-exposed BSW cells, comparable to lung cancer H460 cells.

Various adult stem cells and CSC derived from solid tumors and cancer cell lines have been previously identified by an SP phenotype with enriched stem cell activity [30,31,38]. A small subpopulation of SP cells is characterized by their distinct low Hoechst 33342 dye staining, attributable to their high expression of ABCG2 transporter. To assess the SP phenotype of our cell systems, malignant transformed BSW and lung cancer H460 cells were stained with 5 μg/mL of Hoechst 33342 in the presence or absence of 10 μM fumitremorgin C (FTC), a specific inhibitor of ABCG2 transporter. SP cells, which disappear in the presence of FTC, were identified and calculated as a proportion of the pool population ranging from approximately 15% in BSW and H460 cells to less than 1% in the passage-control BC cells (Figure 1C and E). To confirm the renewal or repopulation ability of the identified CSC, we extracted the cells from tumor spheres and SP subpopulation (designated as first-generation cells) and cultured them for 3 weeks under normal adherent conditions before they were reanalyzed. The cells that were derived from the first-generation of both BSW and H460 spheres and SP preserved the ability to form second-generation spheres and SP (Figure 1D and E).

**Figure 2:**
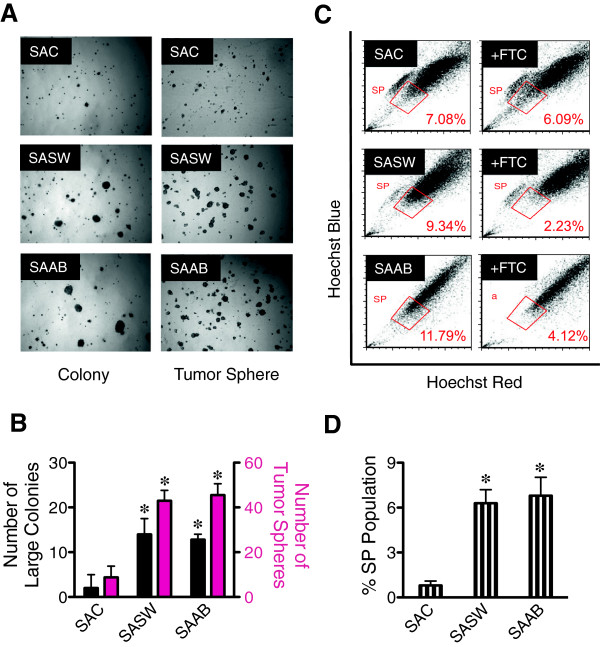
**Chronic exposure to SWCNT induces malignant transformation and CSC formation in small airway epithelial cells. (A)** Analysis of colony formation (*left*) and tumor spheres (*right*) in passage control SAC cells, chronic SWCNT- and asbestos-exposed SASW and SAAB cells after three weeks of culture. **(B)** Quantitative analysis of colony and tumor spheres. **(C)** Analysis of side population (SP) in SAC, SASW, and SAAB cells in the presence or absence of fumitremorgin c (FTC) using FACS. SP cells (*box*) are determined by their disappearance in the presence of FTC. **(D)** Quantitative analysis of SP subpopulation. Data are mean ± SD (*n* = 4). *p < 0.05 *vs*. control SAC cells.

To validate the existence of CSC and SP phenotypes and to rule out the potential effects of SV40 viral proteins as a result of BEAS-2B immortalization, we performed analysis of tumor spheres and SP in chronic SWCNT-exposed small airway epithelial cells (designated as SASW) compared with their passage-control (SAC) cells and positive control asbestos-exposed (SAAB) cells. Consistent with the earlier finding in BSW and H460 cells, SASW cells demonstrated tumor spheres and SP fraction comparable to SAAB cells that are greater than passage-control SAC cells (Figure 2A*-*D), thus supporting the generality of the observed CSC and SP phenotypes in SWCNT-transformed lung cells independent of their SV40 status. To our knowledge, this is the first demonstration of the induction of CSC by chronic low-dose exposure to SWCNT or any other nanomaterials.

### Isolation and characterization of SWCNT-derived CSC cells

**Figure 3:**
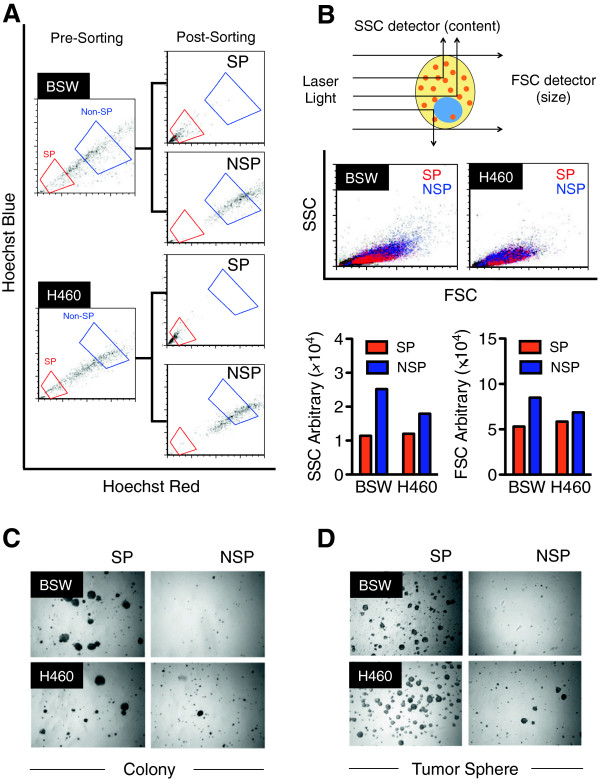
**Isolation and characterization of CSC-like cells from SWCNT-exposed lung epithelial cells. (A)** SP and non-SP (NSP) FACS sorting profiles of malignant transformed BSW and lung cancer H460 cells. Due to their highly dynamic nature of CSC, cells were freshly isolated prior to each experiment. **(B)** Light scattering properties of SP (*red*) and NSP (*blue*) cells derived from BSW and H460 cells as evaluated by flow cytometry. **(C, D)** Analysis of colony formation **(C)** and tumor spheres **(D)** in SP and NSP cells derived from BSW and H460 cells after two weeks of culture.

Flow cytometry-based fluorescence-activated cell sorting (FACS) enables the isolation of SP from chronic SWCNT-exposed cells and positive control lung cancer cells. SP and non-SP (NSP) cell sorting profiles of malignant transformed BSW and lung cancer H460 cells with more than 80% sorted purity are shown in Figure 3A. Sorted SP and NSP cells displayed different light-scattering properties under flow cytometric analysis. Forward-scattered light (FSC) measures mostly diffracted light and is proportional to cell size, whereas side-scattered light (SSC) measures mostly refracted light and is proportional to cell granularity or content [39]. Sorted SP cells derived from both BSW and H460 cells exhibited lower FSC and SSC compared to NSP cells (Figure 3B), suggesting that SP cells are smaller than NSP cells and have lower cytosolic content. The average cell size of SP and NSP cells as measured by Countess® automated cell counter was 13.40 ± 0.7 *vs.* 16.05 ± 0.90 μm in BSW-SP and NSP cells, and 13.03 ± 0.43 *vs*. 14.55 ± 1.06 μm in H460-SP and NSP cells, respectively. These results support the notion that sorted SP and NSP cells are not identical in cellular morphology and structure, and that SP and NSP cells may possess different cellular behaviors. Due to the high dynamics of CSC that are continuously undergoing cell differentiation in normal culture conditions, cells were freshly isolated prior to each experiment.

We next examined the neoplastic growth of SP and NSP cells in soft agar cultures. Figure 3C shows that SP cells derived from both malignant transformed BSW and lung cancer H460 cells were proficient in colony formation, whereas NSP cells grew minimally. To further investigate whether SP cells are enriched with stem cell activity, we cultured the sorted cells under stem cell-selective conditions using tumor sphere formation assays. Figure 3D shows that SP cells formed faster and larger tumor spheres as compared to NSP cells, indicating the enrichment of CSC phenotype in the SP fraction derived from BSW and H460 cells. Having validated that SP cells possess CSC properties, SP phenotype was subsequently used to isolate CSC cells from chronic SWCNT-exposed lung cells and lung cancer cells.

### Aggressive tumor phenotypes of SWCNT-derived CSC cells

**Figure 4:**
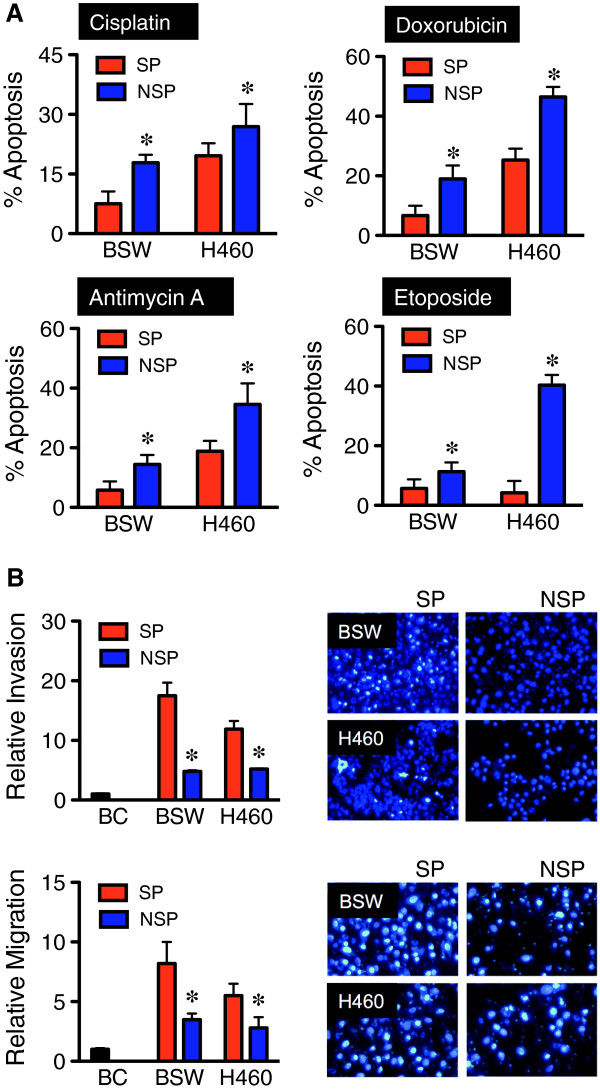
**Aggressive tumor phenotypes of CSC-like cells derived from SWCNT-exposed lung epithelial cells. (A)** Acquired apoptosis resistance of SP cells derived from malignant transformed BSW and lung cancer H460 cells to chemotherapy cisplatin (100 μM), doxorubicin (1 μM), antimycin A (30 μM), and etoposide (500 nM), compared to their respective non-SP (NSP) cells, as analyzed by Hoechst 33342 assay at 24 hours post-treatment. **(B)** Cell invasion (*top*) and migration (*bottom*) assays of SP and NSP cells derived from BSW and H460 cells, and the passage control BC cells, at 48 hours after incubation. Plots are relative invasion and migration level normalized to passage control BC cells. Representative fluorescence micrographs of invading and migrating cells are shown (*right*). Data are means ± SD (*n* = 4). *p < 0.05 *vs.* SP cells.

CSC are known to contribute to the death resistance and aggressive behavior of human cancer cells [40]. We investigated the tumor-associated phenotypes of CNT-derived CSC-like (SP) cells by first measuring their acquired apoptosis resistance to chemotherapy, which is a key characteristic of CSC [41]. Sorted SP and NSP cells derived from malignant transformed BSW and lung cancer H460 cells were treated with various chemotherapeutic agents, including cisplatin, doxorubicin, antimycin A and etoposide, and analyzed for apoptosis by Hoechst 33342 assay at 24 hours post-treatment. Cells having intensely condensed and/or fragmented nuclei were considered apoptotic. Figure 4A demonstrated the resistance of SP cells from both BSW and H460 cells to all chemotherapeutic agents tested *vs*. their matching NSP cells. We further compared their invasion and migration activities, which are key determinants of tumor progression [42] using Transwell® invasion (inserts coated with Matrigel®) and migration (control inserts) assays. The SP cells derived from malignant transformed BSW and lung cancer H460 cells exhibited significantly higher invasion and migration rates than those of the NSP cells (Figure 4B). These results indicate the aggressive tumor phenotype of CSC-like (SP) cells derived from chronic SWCNT-exposed cells, comparable to that of the SP cells derived from lung cancer H460 cells.

### SWCNT-derived CSC cells induce tumor formation *in vivo*

**Figure 5:**
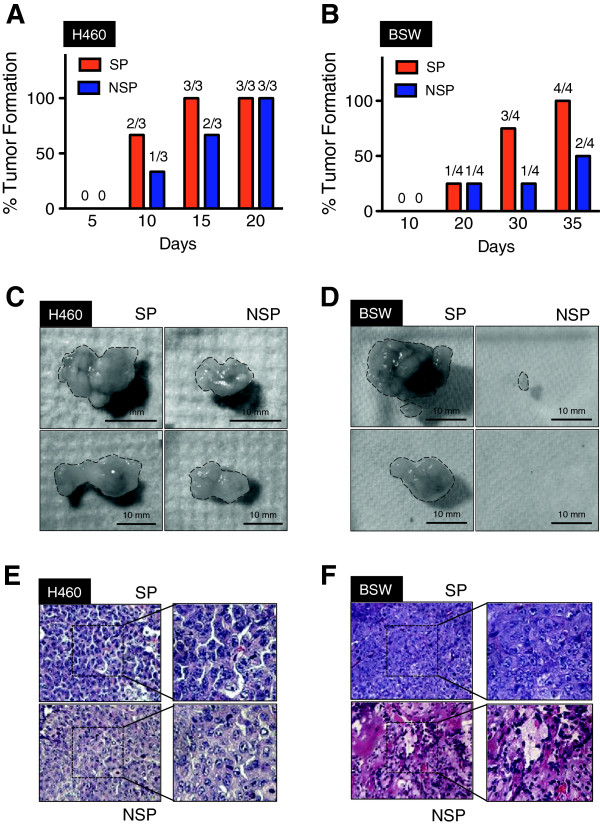
**Tumorigenicity of SWCNT-induced CSC-like cells. (A, B)** Growth kinetics of SP and non-SP (NSP) tumors derived from lung cancer H460 **(A)** and malignant transformed BSW **(B)** cells. **(C, D)** Representative SP (*left*) and NSP (*right*) tumors derived from H460 **(C)** and BSW **(D)** cells. **(E, F)** Representative H&E micrographs of SP and NSP tissue samples derived from H460 **(E)** and BSW **(F)** cells. Condensation of heterochromatin and multi-nucleated cells are evident in the tissue samples from SP and NSP derived from H460, and SP derived from BSW cells.

To test whether the CSC-like (SP) fraction is enriched with tumorigenic cells, the SP and NSP cells from malignant transformed BSW cells and lung cancer H460 cells were subcutaneously injected into NSG mice and monitored for tumor development. At the injection dose of 1 × 10^5^ cells, tumor size (weight) and volume were higher in the SP cells derived from H460 cells but not from BSW cells (Table 2). No tumors were observed in mice injected with passage-control BC cells. As the number of cells is important in tumor initiation [30,31], we performed serial dilution of SP and NSP cells derived from malignant transformed BSW cells. As shown in Table 2, the SP cells from BSW (BSW-SP cells) were able to form tumors in mice with as little as 5 × 10^3^ cells at 25-day latency period. Greater tumor incidence, size, and volume were observed with SP cells than NSP cells at these lower cell numbers. The growth kinetics of SP and NSP tumors from H460 at 1 × 10^5^ (Figure 5A) and BSW at 5 × 10^3^ (Figure 5B) further demonstrated the increased degree of tumor formation of SP cells. Representative SP and NSP tumors of BSW and H460 cells are shown in Figure 5C and D. Histological analysis of SP tumors from BSW showed classical cancer cell morphology including the condensation of heterochromatin and the presence of multinucleated cells, an indicator of mitotic dysfunction [43] (Figure 5F), similarly to those obtained from H460 (Figure 5E). These results demonstrate the tumorigenicity of SWCNT-induced CSC-like (SP) cells and provide a mechanistic insight into the role of CSC in SWCNT tumorigenesis.

**Table 2:** CSC-like (SP) cells are enriched with tumorigenic cells

**Cell types**	**Cell number**	**Incidence**	**Latency (day)**	**Necropsy (day)**	**Tumor**	
					**Volume (mm**^**3**^**)**	**Weight (g)**
**H460**						
SP	1 × 10^5^	3/3	12	21	506 ± 190	0.61 ± 0.28
NSP	1 × 10^5^	3/3	14	21	202 ± 74	0.33 ± 0.13
**BSW**						
SP	1 × 10^5^	3/3	14	21	170 ± 155	0.34 ± 0.21
NSP	1 × 10^5^	3/3	14	21	145 ± 21	0.34 ± 0.24
**BSW**						
SP	5 × 10^4^	2/2	18	28	340 ± 18	0.53 ± 0.47
NSP	5 × 10^4^	1/2	-	28	140 ± 199	0.20 ± 0.28
**BSW**						
SP	5 × 10^3^	4/4	25	35	823 ± 556	1.04 ± 0.64
NSP	5 × 10^3^	2/4	-	35	198 ± 376	0.43 ± 0.51
**BC**	1 × 10^5^	0/2	-	-	-	-

Data are means ± SD (*n* = 2, 3 or 4).

### Expression of putative stem cell markers in SWCNT-derived CSC cells

**Figure 6:**
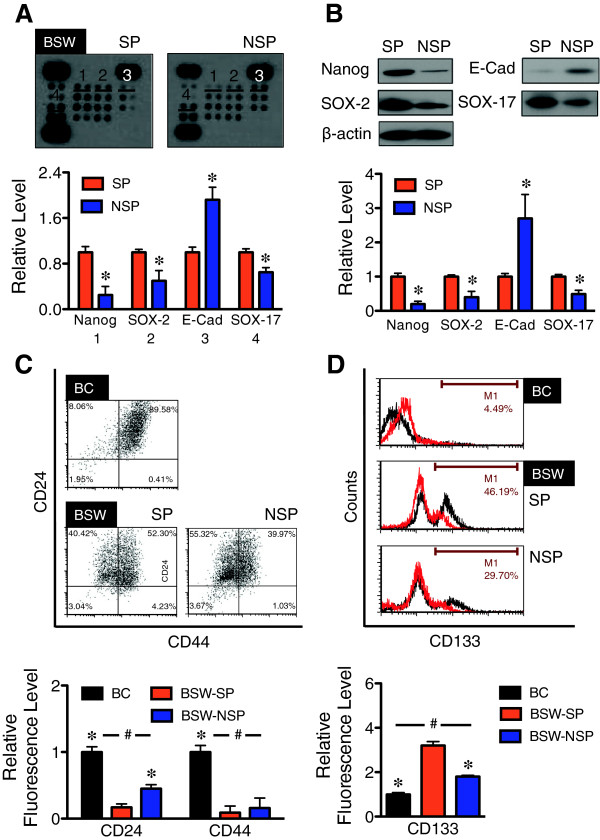
**Expression of stem cell markers in CSC-like cells derived from SWCNT-exposed lung epithelial cells. (A)** Analysis of pluripotent stem cell markers in SP and non-SP (NSP) derived from malignant transformed BSW cells using the Proteome Profiler™ Array (R&D Systems). Pixel densities of remarkable stem cell markers (1) Nanog, (2) SOX-2, (3) E-cadherin (E-Cad), and (4) SOX-17 from the arrays are shown. **(B)** Western blot analysis of Nanog, SOX-2, E-cadherin, and SOX-17 comparing SP and NSP cells derived from BSW cells. **(C, D)** Analysis of stem cell surface markers CD24, CD44, and CD133 in SP and NSP cells derived from BSW cells using flow cytometry, comparing with the passage control BC cells. Plots are relative fluorescence level normalized to control BC cells. Data are means ± SD (*n* = 3). *p < 0.05 *vs.* SP cells, ^#^*p* < 0.05 *vs.* BC cells.

CSC have phenotypic resemblance to normal stem cells. We thus evaluated the possible correlation of putative stem cell markers to CNT-derived CSC using human stem cell protein arrays, which detect several putative stem cell markers including Oct-3/4, Nanog, SOX2, E-cadherein, α-fetoprotein, GATA-4, HNF-3β/FoxA2, PDX-1/IPF1, SOX17, Otx2, TP63/TP73L, goosecoid, snail, VEGF R2/KDR/Flk-1, and HCG. The result, which is shown in Figure 6A and is confirmed by Western blot analysis in Figure 6B, revealed the specific overexpression of Nanog, SOX2 and SOX17, and repression of E-cadherin in SP cells as compared to NSP cells. As putative stem cell markers typically determine self-renewal efficiency and maintenance of stem cells, this data strengthens the stem properties of CSC-like (SP) cells in chronic SWCNT-exposed lung cell population.

Stem cell surface markers have provided powerful tools in stem cell research, i.e., in isolation and characterization of stem cell population and differentiation. As CSC are known to be important in the initiation and progression of cancers, stem cell markers induced by SWCNT might potentially be used for risk assessment and early detection of SWCNT carcinogenic potential. To identify the candidate CSC markers, SP and NSP cells from malignant transformed BSW cells were analyzed for stem cell surface markers CD24, CD44, and CD133 [44,45] in comparison to their passage-control BC cells. A significant decrease in CD24 expression was observed in SP and NSP cells as compared to BC cells with the rank order of expression being BC > NSP > SP (Figure 6C). In contrast, CD133 expression was highest in the SP cells followed by NSP and BC cells (SP > NSP > BC) (Figure 6D). No significant difference in CD44 level was observed in SP and NSP cells, although the level is elevated in BC cells. The correlation between CD24^low^ and CD133^high^ and the tumorigenic activity of SP cells suggests the potential utility of these stem cell markers as candidate biomarkers for the detection of SWCNT tumorigenesis.

### Role of tumor suppressor p53 in SWCNT-derived CSC cells

**Figure 7:**
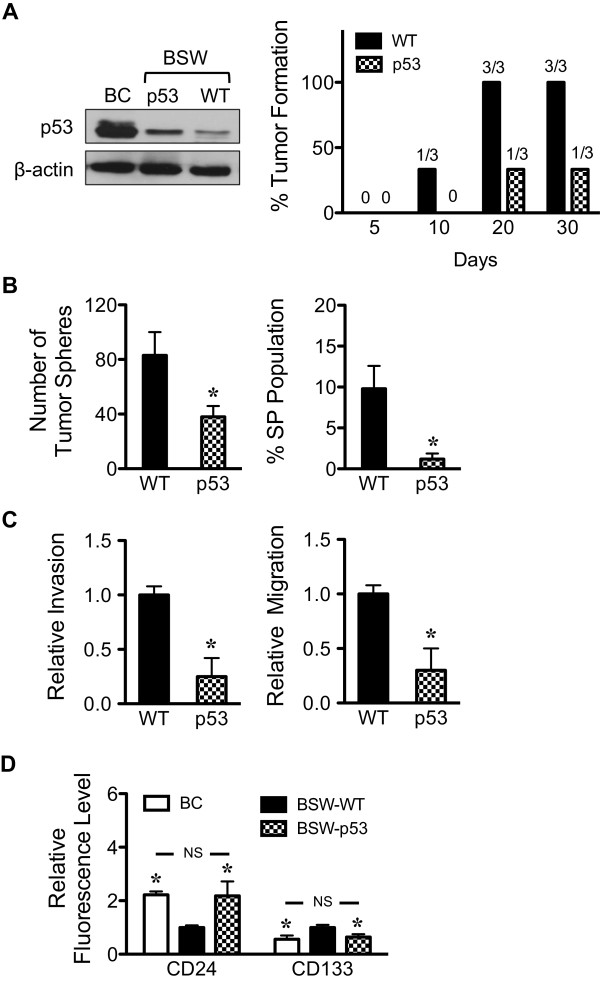
**p53 regulates CSC-like cells from SWCNT-exposed lung epithelial cells. (A)** Western blot analysis of p53 protein (*left*) and tumor growth kinetics (*right*) of wild-type (WT) and p53-overexpressed BSW tumors. **(B)** Quantitative analysis of tumor spheres (*left*) and side population (*right*) in WT and p53-overexpressed BSW cells. **(C)** Cell invasion (*left*) and migration (*right*) assays of WT and p53-overexpressed BSW cells. Plots are relative invasion and migration level normalized to WT cells. **(D)** Analysis of stem cell surface markers CD24 and CD133 in WT and p53-overexpressed BSW cells using flow cytometry. Plots are relative fluorescence level normalized to WT cells. Data are means ± SD (*n* = 3). *p < 0.05 *vs.* WT cells. NS, not significant *vs.* BC cells.

We earlier reported that tumor suppressor p53 mediated the acquired apoptosis resistance of chronic CNT-exposed lung cells, which could be a potential mechanism underlying SWCNT tumorigenesis [24]. However, supporting evidence is lacking. Malignant transformed BSW cells were transfected with p53 or control GFP plasmid, then subcutaneously injected into NSG mice and monitored for tumor formation. At the injection dose of 1 × 10^5^ cells, p53-overexpressing BSW cells showed a remarkably lower incidence of tumor formation as compared to wild-type cells (Figure 7A). To investigate whether p53 could possibly regulate SWCNT-derived CSC, p53-overexpressing cells were evaluated for CSC features of tumor sphere formation and SP subpopulation. Figure 7B shows that p53 restored expression substantially inhibited tumor spheres and SP, indicating the role of p53 in CSC regulation. We further observed the inhibitory effect of p53 on aggressive cancer phenotype, e.g. cell invasion and migration (Figure 7C). Importantly, p53-overexpressing cells were shown to express CD24^high^ and CD133^low^ stem cell surface markers (Figure 7D). These results are in good agreement with the observed lower fraction of CSC and indicate the tumor suppressing activity of p53 in SWCNT tumorigenesis in part through CSC inhibition.

## Discussion

Lung cancer is the leading cause of cancer death, and environmental and occupational exposure is the major cause of most cases [46,47]. The objective of this study was to address human lung cancer risks associated with chronic pulmonary exposure to SWCNT through the fundamental understanding of cellular and molecular processes leading to carcinogenesis. Long-term exposure to workplace-relevant doses of SWCNT, one of the major forms of engineered CNT, were previously shown by our group to induce irreversible malignant transformation and alter cancer-related canonical pathways of lung epithelial cells [24,25]. However, detailed understandings of the pathological process are lacking. Accumulating evidence suggests that various solid tumors including brain, breast, bone marrow, prostrate, colon, and lung contain a rare population of CSC that have a high repopulation capacity and are a major driving force of tumor initiation and progression [29,30,48]. Hence, identifying these CSC provides a fundamental understanding of the carcinogenic process, particularly at the early developmental stage. We investigated whether chronic SWCNT exposure can induce lung CSC and studied their role in tumorigenesis. SWCNT were a focus of this study because they are generally more toxic than MWCNT as indicated by their cytotoxicity [49-51] and fibrogenicity [7,52]. Non-small cell lung cancer H460 cells were used in parallel and served as a positive control. Our results demonstrated for the first time the existence of CSC subpopulation within all clones of chronic SWCNT-exposed lung cells. These cells have the propensity to form tumor spheres under serum-starved, non-adherent conditions (Figures 1 and 2), similar to that observed in non-small cell lung cancer cells and chronic asbestos-exposed lung cells. We also demonstrated the presence of side population (SP), the subpopulation found in various solid tumors with stem cell properties, in the SWCNT-transformed cells. The proportion of SP population correlated well with the tumor sphere-forming capability of CSC. These results suggest that SWCNT can directly interact with lung epithelial cells to initiate CSC formation.

To substantiate the functional role of CSC in SWCNT tumorigenesis, we further characterized and isolated SWCNT-transformed cells into two subgroups based on their SP phenotype as CSC (SP) and non-CSC (non-SP or NSP). The SP phenotype was used because of their demonstrated CSC properties and their reliability of isolation by fluorescence-activated cell sorting (Figure 3). Carcinogenesis involves several cellular processes that contribute to the aggressive behaviors of cells including abnormal growth, increased migration and invasion, evasion of apoptosis, and angiogenesis [53,54]. CSC are known to contribute to the aggressive behaviors of human cancer cells [41,55]. SWCNT-derived CSC acquired apoptosis-resistant and invasive phenotypes as compared to non-CSC (Figure 4). Acquired apoptosis resistance is a hallmark of cancer cells [53]. It promotes cell survival during the carcinogenic process against endogenous anti-growth signals and immune cell killing mechanisms [56]. Whereas, increased cell migration and invasion are crucial to metastasis and are key determinants of tumor progression [42,57]. The results of this study demonstrated the aggressiveness of SWCNT-derived CSC and their ability to evade apoptosis, consistent with the findings observed in CSC derived from solid tumors.

CSC possess high tumorigenic potential [30,31,48]. We found that SWCNT-derived CSC, when injected into mice, have a high tumor-initiating capability as compared to non-CSC (Figure 5). A small number of CSC (e.g. 5 × 10^3^ cells) can induce tumors in mice, whereas non-CSC failed to form tumors in the majority of mice. The presence of a small number of CSC in a large population of non-CSC substantiated the role of CSC as tumor-initiating cells. This is significant because the early stage of carcinogenesis will most likely depend on these tumor-initiating cells.

Our stem cell protein array studies indicate an aberrant expression of several stem cell markers, notably Nanog, SOX2, SOX17, and E-cadherin, in CNT-derived CSC (Figure 6). Hyperactivation of Nanog has previously been shown to promote CSC phenotypes in colon and prostate cancer cells and confer their resistance to apoptosis [58,59]. In breast cancer cells, SOX17 is distinctly upregulated in CSC-like (SP) cells in conjunction with the activation of Wnt/β-catenin signaling pathway [29]. The role of SOX2 in CSC was investigated by several studies. For example, SOX2 overexpression was found to be critical in maintaining the antiapoptotic activity and tumorigenicity of lung CSC, possibly through the upregulation of oncogenes such as c-Myc, Wnt1, Wnt2, and Notch1 [60,61]. Meanwhile, repression of E-cadherin induces epithelial-mesenchymal transition (EMT), the process that is known to involve CSC acquisition in various cancers including lung cancer [62]. Altogether, our studies identify the stem properties of SWCNT-derived CSC and suggest the potential mechanisms that drive CSC acquisition in chronic SWCNT-exposed lung cells.

Stem cell surface markers are powerful tool for characterization and isolation of normal and cancer stem cells. Having demonstrated that SWCNT-induced CSC are crucial to tumor initiation (Figure 5), stem cell surface markers induced by SWCNT could possibly be used for risk assessment of SWCNT carcinogenicity and early detection of SWCNT-induced tumorigenesis. We analyzed surface expression of key biomarkers CD24, CD44, and CD133 in CNT-derived CSC in comparison to non-CSC and non-CNT-exposed control cells. Our results showed the correlation between CD24^low^ and CD133^high^ and the tumor-initiating capability of the cells (Figure 6). CD24^low^ is often used in conjunction with CD44^high^ to identify CSC from breast tumors [63]. However, CNT-derived CSC exhibited a low level of CD44 expression. Our results are in good agreement with a previous report showing CD24^low^ and variable levels of CD44 ranging from high to undetectable in lung CSC [62]. The role of CD133, also known as AC133 or prominin-1, has been reported in lung cancer. CD133-positive subpopulation derived from lung cancer cell lines and patient-derived primary tumors were shown to possess biological features of CSC, including self-renewal and tumor-initiating capabilities [64,65]. In addition, the clinical importance of CD133 was recently shown to correlate with the pathological stage and prognosis of NSCLC patients [45]. With regards to its regulation, it was suggested that CD133 induction was mediated through Oct3/4 and SOX2 under the tumor microenvironment of hypoxia [66]. We also observed a striking upregulation of SOX2 in SWCNT-derived CSC (Figure 6), suggesting the possible mechanism of CD133 induction in these cells.

Most human cancers have a tumor suppressor p53 inactivation [67]. Likewise, our results indicate an important role of p53 in SWCNT tumorigenesis. Chronic SWCNT-exposed BSW cells showed a substantially lower p53 level compared to their passage control cells. Restored expression of p53 to BSW cells results in tumor inhibition, which correlates well with the observed lower CSC fraction (Figure 7). Such restored expression has profound reversal effects on the aggressive cancer phenotypes and stem cell surface markers. These results support the notion that p53 suppresses SWCNT tumorigenesis in part through CSC inhibition and validate the findings that stem cell markers CD24^low^ and CD133^high^ are closely associated with SWCNT tumorigenesis.

**Figure 8:**
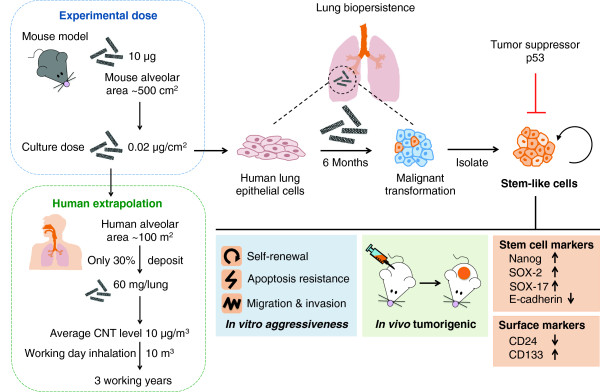
**Schematic diagram representing the overall effects of SWCNT on tumorigenesis.** (*Left*) Extrapolation of CNT dose in mouse and cell culture models to human exposure scenarios in the workplace. (*Right*) Lung biopersistence of SWCNT leads to their chronic interaction with lung epithelial cells and subsequent induction of CSC-like cells. Functional assays showing aggressive cancer phenotypes and tumorigenicity of CSC-like cells.

In conclusion, we presented new evidence supporting the role of CSC in SWCNT tumorigenesis. Chronic exposure of human lung epithelial cells to SWCNT induces CSC with stem properties including the ability to self-renew and form tumor spheres in non-adhering conditions. These cells also possess aggressive cancer phenotypes and are tumorigenic in a mouse model. A schematic summary of our findings is shown in Figure 8. To our knowledge, this is the first demonstration of CSC induced by SWCNT and its role in tumorigenesis. This study supports the prudent adoption of prevention strategies and implementation of exposure control for SWCNT. Although the precise mechanisms underlying CSC acquisition remain to be further elucidated, tumor suppressor p53 in part plays a role. Furthermore, the hyperactivation of Nanog, SOX2 and SOX17, as well as the repression of E-cadherin seem to be involved. The results of this study also suggest the potential utility of stem cell surface markers CD24^low^ and CD133^high^ for early detection of SWCNT tumorigenesis. The CSC model and biomarkers described in this study may be useful in other carcinogenesis studies.

## Materials and methods

### SWCNT characterization and preparation

SWCNT were obtained from Carbon Nanotechnology (CNI, Houston, Texas) and were purified by acid treatment to remove metal contaminates. Elemental carbon analysis was performed by NIOSH Manual of Analytical Methods (NMAM 5040), whereas trace metal analysis was performed by nitric acid dissolution and inductive coupled plasma-atomic emission spectrometry (ICP-AES, NMAM 7300). The specific surface area was measured at -196°C by the nitrogen absorption-desorption technique (Brunauer Emmet Teller method, BET) using a SA3100 Surface Area and Pore Size Analyzer (Beckman Coulter, Fullerton, CA). The diameter and length distribution of the SWCNT were measured by field emission scanning electron microscopy. SWCNT were dispersed by acetone/sonication method [16,24,32] or by using Survanta® as previously described [25]. For acetone/sonication method, SWCNT were treated with acetone and placed in an ultrasonic bath for 24 h. The dispersed SWCNT were then filtered from the solution using a 20-μm nylon mesh screen followed by a 0.2-μm polytetrafluoroethylene filter, which had been weighed prior to use. After filter collection, the dispersed SWCNT were washed thoroughly with distilled water, weighted, and suspended in phosphate-buffered saline with 2–3 minute sonication (Sonic Vibra Cell Sonicator, Sonic & Material Inc, Newtown, CT, USA). For Survanta® dispersion, 1 mg of SWCNT were dispersed in 1 mL of phosphate-buffer saline (PBS) containing 150 μg/mL of Survanta® (Abbott Laboratories, Abbott Park, IL) using light sonication and were diluted in culture medium to obtain the desired concentration.

### Chemicals and reagents

Crocidolite asbestos (CAS# 12001-28-4) was obtained from the National Institute of Environmental Health Sciences (Research Triangle Park, NC). Hoechst 33342, cis-diamminedichloroplatinum II (cisplatin), etoposide, antimycin A, and antibody against -actin were obtained from Sigma-Aldrich (St. Louis, MO). Doxorubicin was obtained from EMD Biosciences (La Jolla, CA). Human pluripotent stem cell array was obtained from R&D Systems (Minneapolis, MN). Antibodies against Nanog, E-cadherin, and peroxidase-labeled secondary antibody were obtained from Cell Signaling Technology (Boston, MA). Antibodies against SOX2 and SOX17 were obtained from Millipore (Billerica, MA). Antibody against p53 was obtained from Santa Cruz Biotechnology (Santa Cruz, CA). Fluorochrome-conjugated antibodies against human CD24, CD44, and CD133 were obtained from Miltenyi Biotec (Auburn, CA).

### Cell culture

Primary human small airway epithelial cells (SAECs) immortalized with hTERT were kindly provided by Dr. Hei (Columbia University, NY) [68]. SAECs were cultured in SABM medium supplemented with Clonetics SAGM SingleQuots (Lonza, Walkersville, MD) which contain 0.4% v/v bovine pituitary extract, 0.1% insulin, 0.1% hydrocortisone, 0.1% retinoic acid, 1% bovine serum albumin, 0.1% transferrin, 0.1% triiodothyronine, 0.1% epinephrine, 0.1% human epidermal growth factor and 0.1% gentamicin. Human bronchial epithelial BEAS-2B cells were obtained from American Type Culture Collection (ATCC; Manassas, VA). They were cultured in Dulbecco’s modified Eagle medium (DMEM) supplemented with 5% fetal bovine serum (FBS), 2 mM L-glutamine, 100 units/mL penicillin and 100 μg/mL streptomycin. Non-small lung cancer cell (NSCLC)-H460 cells were obtained from American Type Culture Collection (Manassas, VA) and were cultured in RPMI 1640 medium supplemented with 5% FBS, 2 mM L-glutamine, and 100 units/mL penicillin/streptomycin. All cells were maintained in a humidified atmosphere of 5% CO_2_ at 37°C.

### Plasmid and transfection

p53 and control GFP plasmids were obtained from Invitrogen (Carlsbad, CA). Cells were transfected with p53 or GFP plasmid by nucleofection using Nucleofector® (Amexa Biosystems, Cologne, Germany), according to the manufacturer’s instructions. Briefly, cells were suspended in 100 μL of nucleofection solution with 2 μg of plasmid and nucleofected using the device program T020. The cells were then resuspended in 500 μL of complete medium and seeded in 60-mm cell culture dishes. Cells were allowed to recover for 48 hours before each experiment. The efficiency of transfection was determined by using a green fluorescent protein reporter plasmid and was found to be ~80%.

### Chronic SWCNT exposure and derivation of SWCNT-transformed cells

Lung epithelial BEAS-2B and SAEC cells were continuously exposed to low-dose SWCNT (surface area dose of 0.02 μg/cm^2^ or concentration dose of 0.1 μg/mL) in culture for 6 months. The cells were passaged weekly at preconfluent densities using a solution containing 0.05% trypsin and 0.5 mM EDTA (Invitrogen, Carlsbad, CA). SWCNT-exposed BEAS-2B cells were designated as BSW cells, whereas SWCNT-exposed SAEC cells were designated as SASW cells. Parallel cultures grown in SWCNT-free medium with the same background level of dispersant provided passage-matched controls and were designated as BC and SAC cells for the cells originated from BEAS-2B and SAEC cells, respectively. After 6 months of exposure, the cells were cultured in normal complete medium, and their cancer and CSC phenotypes were assessed as described below. Human non-small cell lung cancer H460 cells were used as a positive control in the studies.

### Soft agar colony formation assay

Soft agar assay was performed as previously described with minor modifications [69]. Passage-control BC and SAC cells, and chronic SWCNT-exposed BASW and SASW cells (3 × 10^4^ cells) were mixed with culture medium containing 0.5% agar to a final agar concentration of 0.33%. Cell suspensions were immediately plated onto dishes coated with 0.5% agar in culture medium. Colonies were examined under a light microscope after 2 weeks of culture.

### Tumor sphere assay

Tumor sphere assay was performed under non-adherent and serum-free conditions as previously described as stem cell-selective conditions [31,36]. Briefly, cells were resuspended in 0.8% methylcellulose (MC)-based serum-free medium (Stem Cell Technologies, Vancouver, Canada) supplemented with 20 ng/mL epidermal growth factor (BD Biosciences, San Jose, CA), basic fibroblast growth factor and 4 mg/mL insulin (Sigma) and plated at 5 × 10^3^ cells (BC, BSW, and H460) or 1 × 10^4^ cells (SAC and SASW) in ultralow adherent 24-well plates (Corning, Corning, NY). Cells were cultured for two or three weeks. In order to assess the self-renewing property of cells, spheres were collected by gentle centrifugation, dissociated into single cell suspensions, filtered and cultured under conditions described above (second spheres).

### Side population analysis and isolation

Cells were detached by trypsinization and 1 × 10^6^ cells were labeled with 5 μg/mL of Hoechst 33342 in DMEM-F12 medium containing 2% FBS in the presence or absence of 25 μM ABCG2 inhibitor fumitremorgin C (FTC; EMD Biosciences, San Diego, CA) at 37°C for 90 minutes. The cells were then centrifuged and resuspended in ice-cold Hank’s buffer salt solution (HBSS). SP analysis and sorting were performed using BD FACSAria fluorescence-activating (flow cytometry)-based cell sorter (BD Biosciences). The Hoechst dye was excited with a UV laser and its fluorescence was measured with both a 450/20 filter (Hoechst Blue) and 675 LP filter (Hoechst Red). SP fraction was calculated based on the disappearance of SP cells in the presence of FTC using the formula: SP percentage in the absence of FTC - SP percentage in the presence of FTC.

### Apoptosis assay

Apoptosis was determined by DNA condensation/fragmentation assay using Hoechst 33342 dye. Cells were incubated with 10 μg/mL of Hoechst 33342 for 30 minutes and visualized under a fluorescence microscope (Leica Microsystems, Bannockburn, IL). Cells having intensely condensed and/or fragmented nuclei were considered apoptotic. Approximately 1000 nuclei from 10 random fields were analyzed for each sample. The apoptotic index was calculated as the percentage of cells with apoptotic nuclei over total number of cells.

### Cell migration and invasion assays

*In vitro* cell migration and invasion were determined using a 24-well Transwell® unit with polycarbonate (PVDF) filters (8-μm pore size). The membrane was coated with Matrigel® (BD Biosciences, NJ) for the invasion assay, while control inserts were used for the migration assay. Briefly, cells at the density of 3 × 10^4^ cells per well (invasion) or 1.5 × 10^4^ cells per well (migration) were seeded into the upper chamber of the Transwell® unit in serum-free medium. The lower chamber of the unit was filled with a normal growth medium containing 5% FBS. Chambers were incubated at 37°C in a 5% CO_2_ atmosphere for 48 hours. The non-migrating or non-invading cells were removed from the inside of the insert with a cotton swab. Cells that migrated or invaded to the underside of the membrane were fixed and stained with 10 μg/mL Hoechst 33342 for 30 minutes. Inserts were visualized and scored under a fluorescence microscope (Leica DM, IL).

### Xenograft mouse model

Animal care and experimental procedures described in this study were performed in accordance with the Guidelines for Animal Experiments at West Virginia University with the approval of the Institutional Animal Care and Use Committee (IACUC #12-0502). Immunodeficient NOD/SCID gamma mice, strain NOD.Cg-Prkdc^scid^ Il2rg^tm1Wjl^/SzJ (NSG; Jackson Laboratory, Bar Harbor, ME), were maintained under pathogen-free conditions within the institutional animal facility. Food and tap water were given ad libitum. Mice were subcutaneously injected with 5 × 10^3^ - 1 × 10^5^ sorted SP and NSP cells derived from the transformed BSW, positive control H460 cells, or passage-control BC cells suspended in 100 μL of ExtraCel® hydrogel (Advanced BioMatrix, San Diego, CA). Mice were inspected daily for any signs of distress such as weight loss, hunching, failure to groom, and red discharge from the eyes. Tumor growth was monitored daily and tumor size was measured at 21, 28 and 35 days post-injection by using an external caliper (VWR International, Batavia, IL). Tumor volume was calculated using the formula: tumor volume [mm^3^] = 1/2 (length [mm]) × (width [mm]^2^ ). At the end of experiments, mice were euthanized and tumors were dissected and weighted.

### Tumor histopathology

Tumor samples from each tumor were formalin-fixed and paraffin-embedded. Tumor specimens were cut into 5-μm sections and stained with hematoxylin and eosin (H&E) to define the morphology and cellular structure within the tumor region. All tissue sectioning and staining were performed at the West Virginia University Pathology Laboratory for Translational Medicine. The presence of multinucleated cells and condensation of heterochromatin (hematoxylin staining) were considered as cancer-specific patterns.

### Human stem cell array

The Proteome Profiler™ array of human pluripotent stem cell array was commercially obtained from R&D Systems (Minneapolis, MN) and was used according to the manufacturer’s instruction. Briefly, a total of 150 μg of protein lysates were incubated overnight with nitrocellulose membranes dotted with duplicate spots for 15 stem cell markers and control antibodies. Bound proteins were detected with horseradish peroxidase (HRP)-conjugated antibodies using a chemiluminescence detection system (Amersham Biosciences, Piscataway, NJ) and quantified using analyst/PC densitometry software.

### Western blot analysis

After specific treatments, cells were incubated in lysis buffer containing 20 mM Tris–HCl (pH 7.5), 1% Triton X-100, 150 mM NaCl, 10% glycerol, 1 mM Na_3_VO_4_, 50 mM NaF, 100 mM phenylmethylsulfonyl fluoride, and a commercial protease inhibitor mixture (Roche Molecular Biochemicals, Indianapolis, IN) at 4°C for 20 minutes. The lysate was collected and determined for protein content using the Bradford method (Bio-Rad Laboratories, Hercules, CA). Proteins (40 μg) were resolved under denaturing conditions by 7.5-12% sodium dodecyl sulfate-polyacrylamide gel electrophoresis (SDS-PAGE) and transferred onto nitrocellulose membranes (Bio-Rad). The transferred membranes were blocked for 1 hour in 5% nonfat dry milk in TBST (25 mM Tris–HCl, pH 7.4, 125 mM NaCl, 0.05% Tween 20) and incubated with the appropriate primary antibodies at 4°C overnight. Membranes were washed twice with TBST for 10 minutes and incubated with HRP-coupled isotype-specific secondary antibodies for 1 hour at room temperature. The immune complexes were detected by an enhanced chemiluminescence detection system and quantified using analyst/PC densitometry software.

### Stem cell surface marker analysis

Cells were detached by trypsinization and 2 × 10^5^ cells in 100 μL of FACS buffer were labeled with 10 μL of fluorochrome-conjugated antibodies against CD24, CD44, and CD133 (Miltenyi Biotec) in a dark refrigerator for 15 minutes. The cells were then washed, fixed in 2% paraformaldehyde, and resuspended in FACS buffer for analysis by flow cytometry.

### Statistical analysis

The data represent means ± SD from three or more independent experiments as indicated. Statistical analysis was performed by Student’s t test at a significance level of *p* < 0.05.

## Abbreviations

CNT: Carbon nanotubes; SWCNT: Single-walled CNT; CSC: Cancer stem cells; FACS: Fluorescence-activated cell sorting; SP: Side population; NSP: Non-SP; BC: Passage-matched control bronchial epithelial cells; BSW: Chronic SWCNT-exposed bronchial epithelial cells; SAC: Passage-matched control small airway epithelial cells; SASW: Chronic SWCNT-exposed small airway epithelial cells; SAAB: Chronic asbestos-exposed small airway epithelial cells; SSC: Side-scattered light; FSC: Forward-scattered light; NSG mice: NOD/SCID gamma mice; H&E: Hematoxylin and eosin; E-Cad: E-cadherin; WT: Wild-type.

## Competing interests

The authors declare that they have no potential competing interests.

## Authors’ contributions

SL designed research, carried out molecular and functional assays, animal studies, and prepared the manuscript. LW characterized particles and performed chronic exposure. VC participated in the design of the study and prepared the manuscript. YR conceived and coordinated the project, and prepared the manuscript. All authors read and approved the final manuscript.

## Authors’ information

This work was supported by the NIH Grants R01-HL095579 and R01-ES022968, NSF Grant EPS-1003907, and MBRCC, Sara C. Allen and James F. Allen Comp Lung Cancer Research Fund.
